# Supplementary material for: Patterns of pollen and resource limitation of fruit production in Vaccinium uliginosum and V. vitis-idaea in Interior Alaska
Source: PLoS One. 2020 Aug 19;15(8):e0224056. doi: 10.1371/journal.pone.0224056 (PMC7446802; doi:10.1371/journal.pone.0224056)

Models run with only conspecific TFR

a) *V. uliginosum* high elevation

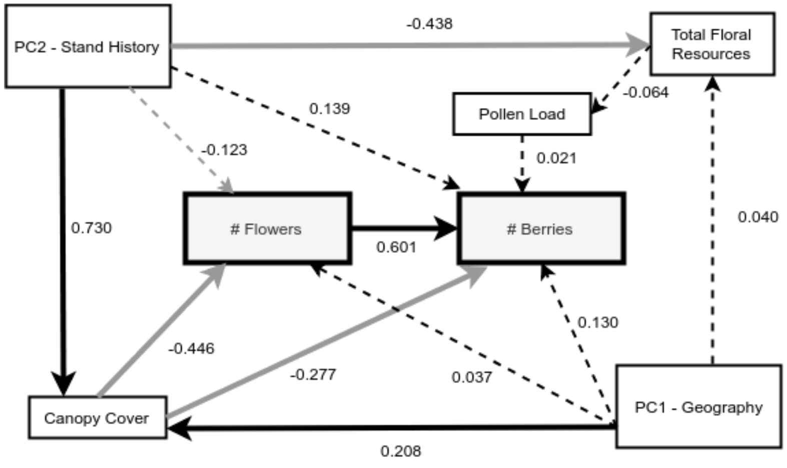

c) *V. vitis-idaea* high elevation

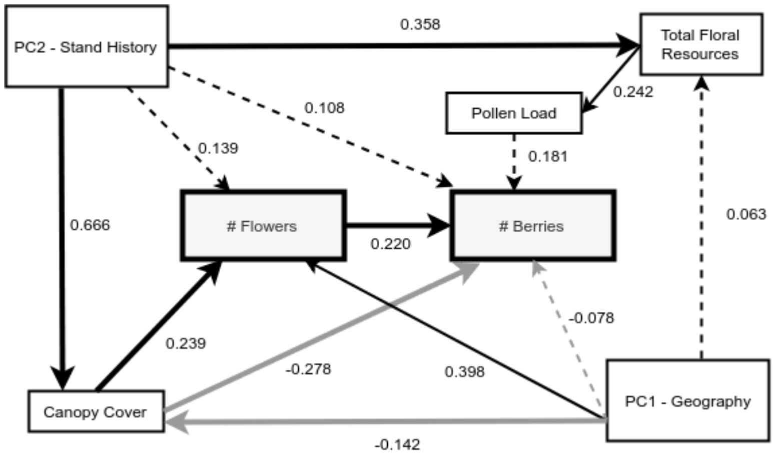

b) *V. uliginosum* low elevation

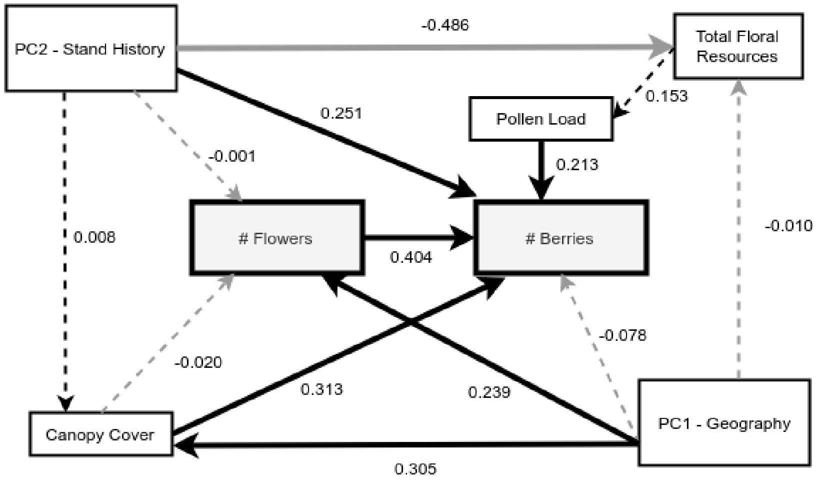

d) *V. vitis-idaea* low elevation

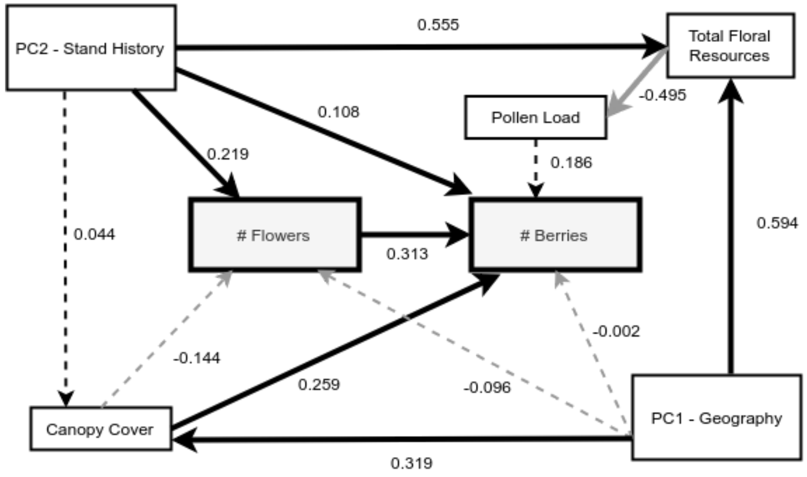

Supplement: S2 Fig — a) high elevation blueberry (Vaccinium uliginosum), n = 80 b) low elevation blueberry, n = 106 c) high elevation lingonberry (V. vitis-idaea), n = 97 d) low elevation lingonberry, n = 98. Grey boxes are the response variables. Solid lines represent significant pathways, while dashed lines are non-significant. Black lines represent positive pathways, while grey lines are negative pathways. Path coefficients are the standardized estimates from the SEM. (PDF) [file pone.0224056.s002.pdf]
