# Supplementary figures and images for: Patterns of pollen and resource limitation of fruit production in Vaccinium uliginosum and V. vitis-idaea in Interior Alaska
Source: PLoS One. 2020 Aug 19;15(8):e0224056. doi: 10.1371/journal.pone.0224056 (PMC7446802; doi:10.1371/journal.pone.0224056)

a) Blueberry upland:  $R^2 = 0.55$

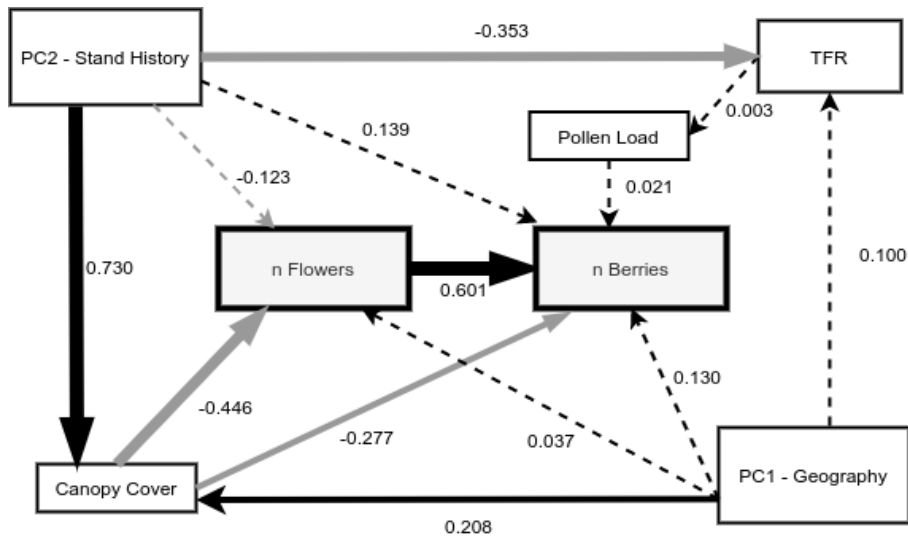

c) Lingonberry upland:  $R^2 = 0.12$

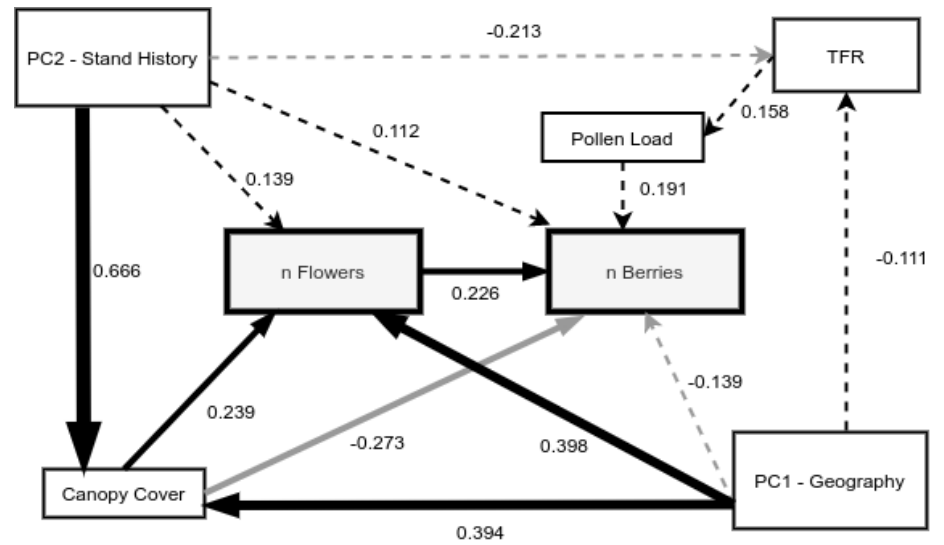

b) Blueberry lowland:  $R^2 = 0.32$

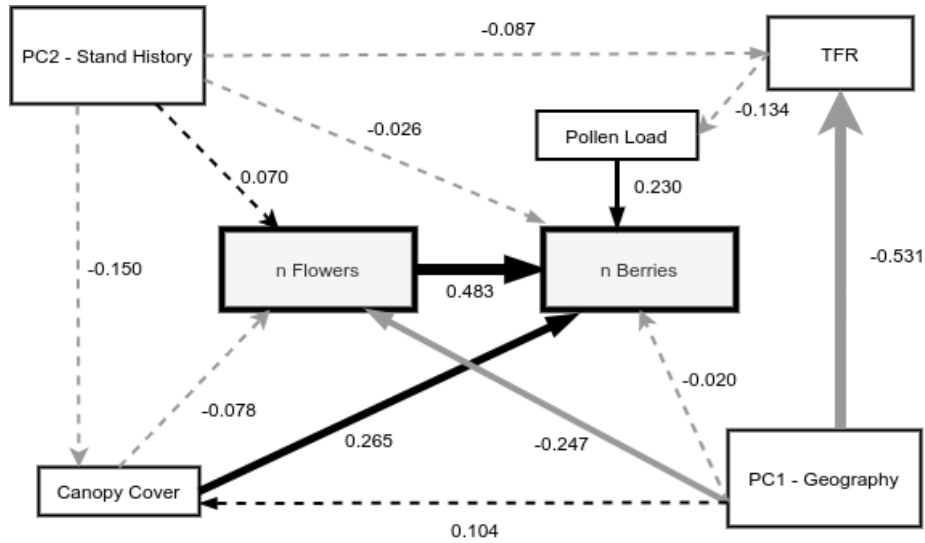

d) Lingonberry lowland:  $R^2 = 0.26$

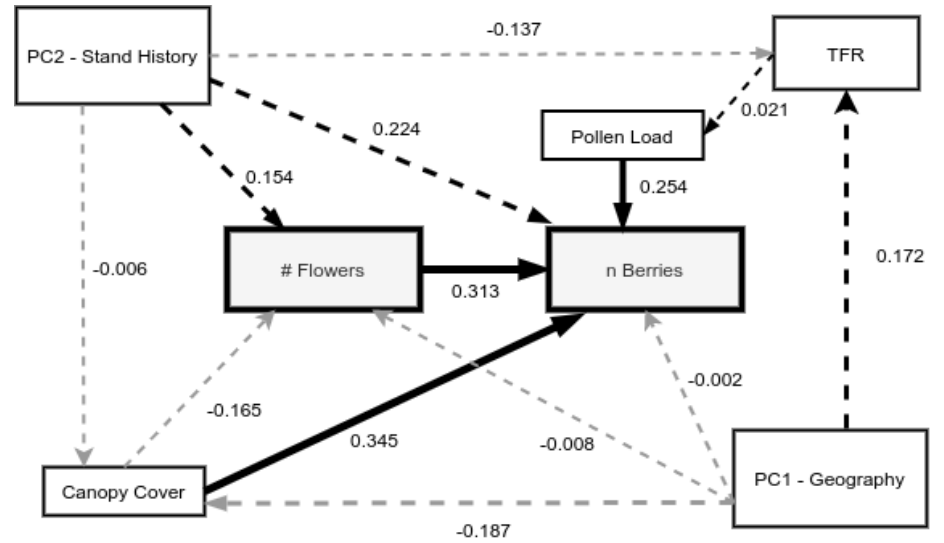

Supplement: S3 Fig — Fit statistics worsened a) high elevation blueberry (Vaccinium uliginosum), n = 80 b) low elevation blueberry, n = 106 c) high elevation lingonberry (V. vitis-idaea), n = 97 d) low elevation lingonberry, n = 98. Grey boxes are the response variables. Solid lines represent significant pathways, while dashed lines are non-significant. Black lines represent positive pathways, while grey lines are negative pathways. Path coefficients are the standardized estimates from the SEM. (PDF) [file pone.0224056.s003.pdf]
