# Supplementary material for: Patterns of pollen and resource limitation of fruit production in Vaccinium uliginosum and V. vitis-idaea in Interior Alaska
Source: PLoS One. 2020 Aug 19;15(8):e0224056. doi: 10.1371/journal.pone.0224056 (PMC7446802; doi:10.1371/journal.pone.0224056)
Supplement: S1 Table — Upland sites are highlighted grey, lowland sites are white. (DOCX) [file pone.0224056.s004.docx]

| Site | Latitude (⁰) | Longitude (⁰) | Elevation  (m) | Age (years) | Soil Moisture | Primary Canopy |
| --- | --- | --- | --- | --- | --- | --- |
| BFY1 | 65.119 | -147.429 | 497 | 13 | Subxeric | Open |
| BFY10 | 65.118 | -147.449 | 425 | 13 | Subxeric/Mesic | Open |
| BFY6 | 65.13 | -147.477 | 210 | 13 | Mesic | Sapling Hardwood |
| GSI1 | 64.8 | -148.413 | 144 | 51 | Mesic | Black Spruce |
| GSI2 | 64.802 | -148.42 | 129 | 51 | Subhygric | Open Black Spruce |
| GSM3 | 64.916 | -147.832 | 188 | 175 | Subhygric | Black Spruce |
| GSM4 | 64.942 | -148.253 | 504 | 85 | Subxeric/Mesic | Black Spruce |
| MDI5 | 64.882 | -148.398 | 215 | 59 | Mesic | Open Black Spruce |
| UP4A | 64.768 | -148.298 | 490 | 100 | Subxeric | Black Spruce |
| UP4B | 64.771 | -148.273 | 400 | 200 | Subxeric | Black Spruce |
| UP4C | 65.153 | -147.491 | 233 | 130 | Subxeric | Black Spruce |
| UP4D | 65.169 | -147.55 | 620 | 100 | Subxeric | Black Spruce |
| WCM1 | 65.124 | -147.973 | 246 | 190 | Subxeric | Open Black Spruce |
| WCM3 | 65.154 | -147.863 | 271 | 190 | Subxeric | Black Spruce |
| WCM4 | 65.167 | -147.894 | 459 | 190 | Subxeric | Black Spruce |
| WDI5 | 65.145 | -148.018 | 477 | 46 | Subxeric | Hardwood |
| WDI6 | 65.148 | -148.026 | 505 | 46 | Subxeric | Hardwood |
