# Supplementary material for: Patterns of pollen and resource limitation of fruit production in Vaccinium uliginosum and V. vitis-idaea in Interior Alaska
Source: PLoS One. 2020 Aug 19;15(8):e0224056. doi: 10.1371/journal.pone.0224056 (PMC7446802; doi:10.1371/journal.pone.0224056)
Supplement: S2 Table — "Geography" refers to PC2 scores and "Stand History" to PC1 scores. "TFR" is total floral resources. Dashes indicate this link was not assessed in the model. (PDF) [file pone.0224056.s005.pdf]

S5 Table. Direct and indirect effects on number of berries in each SEM shown in S2 in order of the absolute value of the total effect.

**Blueberry**	Direct	Indirect	Total	**Lingonberry**	Direct	Indirect	Total
Flowers	0.567	--	0.567	Pollen	0.222	0	0.222
Pollen	0.088	--	0.088	Flowers	0.175	0	0.175
Canopy	0.025	-0.102	-0.078	Stand History	0.131	-0.037	0.094
Geography	-0.139	0.063	-0.076	TFR	--	0.054	0.054
Stand History	0.081	-0.086	0.006	Geography	-0.139	0.019	0.012
TFR	--	-0.005	-0.005	Canopy	-0.028	0.018	-0.011

"Geography" refers to PC2 scores and "Stand History" to PC1 scores. "TFR" is total floral resources. Dashes indicate this link was not assessed in the model.
