# Supplementary material for: Patterns of pollen and resource limitation of fruit production in Vaccinium uliginosum and V. vitis-idaea in Interior Alaska
Source: PLoS One. 2020 Aug 19;15(8):e0224056. doi: 10.1371/journal.pone.0224056 (PMC7446802; doi:10.1371/journal.pone.0224056)
Supplement: S3 Table — (DOCX) [file pone.0224056.s006.docx]

|  | CV Number of Flowers | | CV Number of berries | |
| --- | --- | --- | --- | --- |
|  | Blueberry | Lingonberry | Blueberry | Lingonberry |
| BFY1 | 0.208 | 0.444 | 0.949 | 0.782 |
| BFY10 | 0.553 | 0.550 | 1.160 | 0.695 |
| BFY6 | 0.999 | 0.540 | 2.486 | 1.595 |
| GSI1 | 0.631 | -- | 1.310 | -- |
| GSI2 | 0.923 | 0.853 | 2.663 | 3.464 |
| GSM3 | 1.407 | 0.431 | 2.462 | 1.236 |
| GSM4 | 1.359 | 0.973 | 1.954 | 2.335 |
| MDI5 | 0.724 | 0.532 | 3.464 | 1.031 |
| UP4A | 0.883 | 0.379 | 1.370 | 1.095 |
| UP4B | 1.115 | 0.247 | 0.729 | 0.692 |
| UP4C | 0.726 | 0.480 | 1.605 | -- |
| UP4D | 0.850 | 0.479 | 1.168 | 0.841 |
| WCM1 | 1.415 | 0.675 | 3.464 | -- |
| WCM3 | 1.410 | 0.410 | 2.533 | -- |
| WCM4 | 0.584 | 0.336 | 1.477 | 1.172 |
| WDI5 | 0.849 | 0.487 | 1.841 | 1.642 |
| WDI6 | -- | 0.564 | -- | 1.407 |
|  |  |  |  |  |
| Overall | 0.868 | 0.334 | 1.570 | 0.789 |
